# Gibbs states and Brownian models for coexisting haze and cloud droplets

**DOI:** 10.1126/sciadv.adq7518

**Published:** 2024-11-15

**Authors:** Manuel Santos Gutiérrez, Mickaël David Chekroun, Ilan Koren

**Affiliations:** ^1^ Department of Earth and Planetary Sciences, Weizmann Institute of Science, Rehovot 76100, Israel.; ^2^ Department of Atmospheric and Oceanic Sciences, University of California, Los Angeles, Los Angeles, CA 90095, USA.

## Abstract

Cloud microphysics studies include how tiny cloud droplets grow and become rain. This is crucial for understanding cloud properties like size, life span, and impact on climate through radiative effects. Small weak-updraft clouds near the haze-to-cloud transition are especially difficult to measure and understand. They are abundant but hard to capture by satellites. Köhler’s theory explains initial droplet growth but struggles with large particle groups. Here, we present a stochastic, analytical framework building on Köhler’s theory to account for (monodisperse) aerosols and cloud droplet interaction through competitive growth in a limited water vapor field. These interactions are modeled by sink terms, while fluctuations in supersaturation affecting droplet growth are modeled by nonlinear white noise terms. Our results identify hysteresis mechanisms in the droplet activation and deactivation processes. Our approach allows for multimodal cloud’s droplet size distributions supported by laboratory experiments, offering a different perspective on haze-to-cloud transition and small cloud formation.

## Teaser

Stochastic nonlinear mechanisms explain the diversity of cloud droplet size distributions found in previous experimental results.

## INTRODUCTION

Clouds play a key role in the climate energy balances and in the water cycle (*1*). A large part of the uncertainties in climate predictions is attributed to limitations in our understanding of cloud physics and therefore how to parameterize such clouds in global climate models (*2*). Out of all cloud types, small warm clouds are highly abundant and pose serious challenges for cloud research. Their diminutive size often falls below the resolution limits of Earth-observing satellites, making direct observation difficult. Additionally, their weak optical signatures can hinder detection and measurement (*3*–*5*). These factors contribute to a substantial knowledge gap regarding the role and impact of small warm clouds in the global climate system.

The cloud’s droplet size distribution (DSD) is among the most important microphysical properties. The DSD properties are coupled to all dynamical, turbulent, optical, and stochastic cloud processes. DSDs affect the cloud’s evolution in time, rain processes, cloud depth, size, and lifetime (*6*). When supersaturation is high enough, cloud condensation nuclei (CCNs) are activated into cloud droplets and grow by condensation. This is the main mechanism for droplet growth at the early stages of a warm cloud. If the cloud’s dynamics is weak (shallow clouds) or if the aerosol concentration is high, condensation could be the main growth mechanism throughout the whole cloud’s lifetime as droplets do not become large and varied enough to support efficient autoconversion of droplets by the stochastic processes of collision and coalescence [see chapters 7 and 8 in (*7*)].

Köhler theory provides a framework for understanding the activation of aerosol particles into cloud droplets. In low supersaturation conditions, only a small subset of the aerosol population, typically the largest and most hygroscopic particles, is activated. The activation process is governed by a delicate balance between the Kelvin effect, which opposes activation due to the curvature of the droplet surface, and the Raoult effect, which favors activation due to the presence of dissolved solutes. This interplay is central to Köhler theory (*8*). As a result of this competition, aerosol particles can exist in two distinct thermodynamic states: haze particles and activated droplets (*6*). Haze particles remain typically in stable equilibrium with the surrounding environment, while activated droplets grow by diffusion. However, other microphysics regimes in a turbulent flow can lead to intricate interactions between haze and activated droplets, resulting, e.g., into nonlinear behaviors such as oscillatory pulses of activation at low particle concentrations (*9*).

The mechanisms of activation and deactivation are, however, not symmetric. To activate a cloud droplet, it is necessary to attain a critical level of supersaturation λ_K_, typically well above 0%. When the supersaturation is decreased to λ_K_ during the deactivation process, an activated cloud droplet may remain sufficiently large to continue experiencing diffusion of water vapor onto its surface. This positive feedback mechanism results into a deactivation threshold that is strictly lower than λ_K_. In this case, activation-deactivation exhibits hysteresis, a phenomenon previously documented in the cloud physics literature (*10*, *11*).

From a nonlinear dynamics perspective, such a hysteresis phenomenon results from the presence of two saddle-node (SN) bifurcation curves that meet tangentially [chapter 8.2 in (*12*)]. Recall that a SN bifurcation is a type of bifurcation where two equilibrium points “collide” and disappear as a system’s parameter is varied (here supersaturation). It is at the core of the notion of tipping points (*13*) and occurs in many fields of physics such as combustion theory (*14*, *15*), energy balance climate models (*16*, *17*), or ocean dynamics (*18*–*21*). In the context of cloud physics, the authors in (*11*) have shown that the activation process of a cloud particle following the Köhler condensational growth equation undergoes such a SN bifurcation in which the haze equilibrium merges with an unstable equilibrium. At this bifurcation point, the stability of the haze equilibrium is lost due to this merging. On analytical and numerical grounds, it was shown in (*11*) that hysteresis does occur for air-parcel models accounting for temperature and pressure variations, suggesting the presence of the other SN bifurcation where the activated equilibrium merges with an unstable one. Here, we provide a general framework for hysteresis to take place from the Köhler condensational growth equation alone by incorporating a sink term accounting, e.g., for the evolution of ambient heat and moisture content, or sedimentation effects.

Our approach also accounts for turbulent effects. Clouds in turbulent environments exhibit a complex interplay between microphysical processes and turbulent dynamics. Turbulence strongly influences the life cycle of clouds, as demonstrated in (*22*). Turbulent models are essential for understanding droplet size spectra broadening (*23*, *24*), the mixing of cumulus clouds with their surroundings (*5*, *25*), and the parameterization of mixed-phase clouds in global circulation models (*26*, *27*). In warm clouds, turbulence-induced localized temperature gradients and small-scale fluctuations can lead to supersaturation variations. These variations can activate haze particles even in environments with a mean subsaturation, and similarly, cloud particles may deactivate in saturated conditions if the fluctuations are sufficiently strong (*25*, *28*). Therefore, microphysical fluctuations are crucial for accurately representing droplet size spectra in turbulent cloud environments. We represent these effects by means of stochastic parameterizations involving Brownian motions (*21*, *29*) and possibly depending on the particle’s size.

Thus, this research explores the activation and deactivation of cloud droplets in warm clouds, offering new theoretical perspectives. We aim to find analytical formulas for describing the cloud’s DSDs when both small cloud droplets and haze particles coexist, assuming condensation as the only growth mechanism. To this end, we add a sink term to the classical formulation of Köhler’s equation to parameterize the interaction of a population of droplets or other size-depleting mechanisms, like water vapor consumption or sedimentation (*11*, *30*, *31*). The resulting equation describing condensational growth of droplets composing a monodisperse aerosol population allows for multiple stable states to coexist besides the “haze” state, meaning the system exhibits multistability. Although the haze state remains stable, this multistability suggests that activated droplets can get stuck around a specific size due to the balancing act between two factors: the Köhler instability and mechanisms that decrease supersaturation or remove particles [see also (*10*, *11*, *32*)].

This is where turbulence effects throw a wrench in the picture. The stable states become “fuzzy states” that are encountered statistically (metastable) at various rates of occurrence. This means that particles can switch between activated and deactivated states, similar to how Brownian particles move around in a bumpy landscape. These turbulent jolts also strongly affect the activation-deactivation process into the form of various possible hysteresis loops (see the “Hysteresis effects” section). As a result, droplets can become activated at lower levels of supersaturation compared to the standard Köhler threshold.

Finally, our framework allows us to derive analytical formulas for the DSDs within a cloud volume, accounting for these stochastic turbulent effects. In particular, our stochastic model predicts that the DSDs in the Pi cloud convection chamber experiments behave like Gibbs states, i.e., an equilibrium probability distribution that maximizes the system’s entropy subject to constraints on its average energy like in statistical mechanics (*33*). It is shown that our theoretical predictions based on this formalism closely match the actual DSDs observed in these experiments (see the “Pi-chamber empirical distributions versus Gibbs states” section).

## RESULTS

### Köhler theory

The theory of Köhler is the thermodynamical backbone to explain the formation of cloud droplets out of suspended aerosols in the atmosphere. It establishes the critical relative humidity threshold beyond which suspended particles activate and grow by diffusion (condensation) (*8*). The critical threshold is above saturation (hence, the supersaturation term is used), and it depends on the initial size and chemical composition of the particle in question. These particles are, according to Köhler, present in two distinct/dichotomical thermodynamic states: The first is known as haze and it is observed when the aerosol is humidified, although it remains at equilibrium with its moist environment. The second state is referred to as activated droplet, whereby the particle harvests the available moisture by diffusion and, in principle, grows indefinitely. However, in nature, the available water vapor molecules that are in supersaturation are limited, and in many cases, droplets and haze coexist in the cloud, and particles can oscillate between the two states. This is observed in the case of small warm clouds (*34*, *35*).

**Fig. 1.**
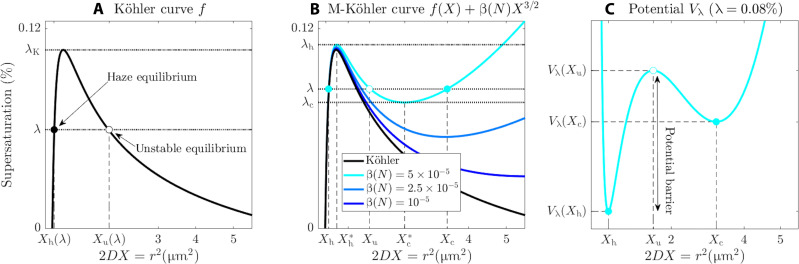
Köhler and M-Köhler curves, and underlying potential. (**A**) Köhler curve associated with NaCl (see Table 1 for the parameter values). A dry radius of 50 nm is prescribed. λ_K_ denotes Köhler’s critical supersaturation and λ, an example of supersaturation. (**B**) In black, the Köhler curve associated with NaCl as (A). The cyan to blue curves show different M-Köhler curves associated with Eq. 6 for different values of the parameter β(*N*) as indicated in the legend. For the cyan curve is shown its intersection with a given supersaturation λ in between λ_c_ and λ_h_. Each filled dot corresponds to a stable equilibrium, while the empty dot corresponds to the unstable equilibrium. (**C**) The potential function associated with the cyan M-Köhler curve for the supersaturation value as indicated in the title. The solid/empty dots locate the stable/unstable equilibria.

The critical relative humidity threshold needed for activation of a given aerosol with a solubility constant *k* and a dry radius *r*_d_ is analytically obtained using Köhler’s equation (*7*, *8*). Here, to simplify some derivations, λ denotes the part of the relative humidity above 100%. If *r*(*t*) denotes the radius of a given droplet at time *t*, its evolution is approximated by (*8*, *11*)

$$\left\{ \begin{array}{c} \overset{\cdot }{r}=\frac{D}{r}\left[\lambda -\lambda_{\text{eq}}\left(r\right)\right]\\ \lambda_{\text{eq}}\left(r\right)=\frac{A}{r}-\frac{B}{r^{3}} \end{array}\right.$$ (1)

where *D* is the diffusivity constant, *A* relates to the water surface tension, $B={\mathrm{kr}}_{d}^{3}$, λ is the ambient supersaturation, and λ_eq_(*r*) determines the equilibrium supersaturation at the surface of a droplet of radius *r*, commonly known as Köhler curve (*36*). Equation 1 shows that the particle is in equilibrium when λ = λ_eq_(*r*). To simplify the proposed model, we replace *r* with the variable *X* = *r*^2^/2*D*, which has time units and could be viewed as relaxation time for diffusion. With this change of variable, Eq. 1 translates into the nondimensional form

$$\left\{ \begin{array}{c} \overset{\cdot }{X}=\lambda -f\left(X\right)\\ f\left(X\right)=A{\left(2\mathrm{DX}\right)}^{-1/2}-B{\left(2\mathrm{DX}\right)}^{-3/2} \end{array}\right.$$ (2)

with *f*(*X*) denoting the equilibrium supersaturation λ_eq_(*r*) as a function of (the scaled squared radius) *X*. A rudimentary analysis of *f*(*X*) reveals a single global maximum located at *X*_K_ = 3*B*/(2*DA*) and with critical supersaturation value λ_K_ = *f*(*X*_K_) = (4*A*^3^/27*B*)^1/2^ [see also chapter 6.1 in (*36*)]. An example of Köhler curve is shown in Fig. 1A, whose parameters are given in Table 1. This curve reaches its maximum at *X*_K_ = 6 × 10^−3^ s or at *r*^2^ = 0.48 μm^2^.

**Table 1.** Köhler curve’s parameters for NaCl.

	*k*	*r*_d_ (μm)	*A* (μm)	*D* (μm^2^ s^−1^)
NaCl	1.28	5 × 10^−2^	10^−3^	40

For a specified dry radius, *r*_d_, a particle will activate into a cloud droplet when the ambient supersaturation, λ, surpasses its critical supersaturation, λ_K_. This activation process occurs through diffusional growth. Conversely, for a given supersaturation less than λ_K_, particles with radii smaller than (2*DX*_u_)^1/2^ will remain in a stable haze state. These equilibrium points are visually represented by the intersection of a horizontal line at the level of λ and the Köhler curve, as depicted in Fig. 1A. The solid and empty dots within this figure denote the stable and unstable equilibria, respectively.

While Köhler theory is established for single particles, clouds consist of large families of particles coexisting in the same humidity environment. When a collection of droplets consumes the available supersaturation, the particles may cease growing by condensation, and the DSDs stagnate and stop being displaced toward larger sizes (*10*, *37*). Here, we modify the Köhler equation to the case of many particles in conditions of small warm clouds, when the available supersaturation is a limit factor. The proposed model extends the condensational growth equation—governed by the Köhler curve—by coupling all particles to supersaturation consumption and by adding stochastic disturbances to account for (micro) small-scale turbulent effects.

### Multistable Köhler curves

In cases with relatively small supersaturation, as particles grow, they may consume the available supersaturation and therefore reduce their growth rate until reaching stagnation. In the realm of single-particle models, these effects can be modeled by the introduction of a sink function into Eq. 2

$$\overset{\cdot }{X}=\lambda -\underset{\;M-K\ddot{o}\text{hler}\;\text{curve}}{\underbrace{\left[f\left(X\right)-g\left(X\right)\right]}}$$ (3)

in which *g* denotes the sink term. The function *f-g* is called an M-Köhler curve hereafter. The term *g* should remain negative and should decrease as *X* increases, as its main role is for accounting for saturation mechanisms in the particle growth. More precisely, this sink term in growth rate aims to (i) model the supersaturation budget and (ii) parameterize other stagnating mechanisms into the condensational growth rate, for instance, due to sedimentation (*31*) or the presence of a thermal inversion layer that reduces supersaturation and stops droplet growth (*35*, *38*).

The sink term can also relate to first-order fluctuations in supersaturation due to condensation. When a sizable amount of particles coexist in the same cloud parcel, the constant supersaturation assumption has to be amended taking account changes in λ in the course of time, assuming λ to be time dependent in the first equation of Eq. 2. At a first order, we are concerned with describing the evolution of λ(*t*) = λ + λ′(*t*) with λ′ (0) = 0, i.e., we are seeking for a model of the rate of change of the fluctuations λ′ around a given value λ.

Following (*11*), the rate of change λ′ can be approximated by the ratio $-{\overset{\cdot }{\rho }}_{v}/\rho_{\text{vs}}$, in which ρ_v_ denotes the ambient vapor density away from the droplet, while ρ_vs_ denotes the vapor density at saturation. Then, by expressing ρ_v_ as ρ_v_ = 4π*N*ρ_w_*r*^3^/3, accounting for the density of liquid water ρ_w_ and the droplet number concentration *N* of identical (monodisperse) particles in the cloud parcel, we arrive at

$$\frac{\;d}{\;dt}\lambda \prime =-\beta \left(N\right)\frac{\;d}{\;dt}\left(X^{\frac{3}{2}}\right)$$ (4)

when written in the variable *X* = *r*^2^/(2*D*), where the constant β(*N*) is given by

$$\beta \left(N\right)=\frac{2^{7/2}D^{3/2}{\pi \rho }_{w}}{3\rho_{\text{vs}}}N$$ (5)

see equations 9 and 10 in (*11*).

By integrating each side of Eq. 4, we obtain λ′(*t*) = −β(*N*)*X*^3/2^. When this expression of λ′ is used in λ(*t*) = λ + λ′(*t*), we arrive at λ(*t*) = λ − β(*N*)*X*^3/2^, which leads, from the first equation in Eq. 2, to the following condensational growth model

$$\overset{\cdot }{X}=\lambda \left(t\right)-f\left(X\right)=\lambda -f\left(X\right)-\beta \left(N\right)X^{3/2}$$ (6)

Thus, the term −β(*N*)*X*^3/2^ acts as the sink function *g* in Eq. 3: It is negative and decreases as *X* increases. For large values of *X*, it saturates the particle growth. A few M-Köhler curves are shown in Fig. 1B for different β(*N*) values.

A careful examination of Eq. 6 reveals that Köhler’s critical supersaturation λ_K_ and radius are modified due to the sink function. The function *f*(*X*) + β(*N*)*X*^3/2^ can now admit a local minimum in addition to the maximum exhibited by the Köhler curve *f*. These are obtained as zeros of the following depressed cubic polynomial equation

$$-3\beta \left(N\right)X^{3}+A{\left(2D\right)}^{-1/2}X-3B{\left(2D\right)}^{-3/2}=0$$ (7)

Sufficient conditions for Eq. 7 to have two positive solutions are easily derivable. Actually, the existence of haze and activated steady states, $X_{h}^{*}$ and $X_{c}^{*}$ (with $X_{c}^{*}\geq X_{h}^{*}$), can be inferred for condensational growth models analogous to Eq. 6 with more general sink functions −β(*N*)*X*^α^ (α > 1) (see Proposition 1 in the “Multistability” section in Materials and Methods). In any event, as shown in Fig. 1B, the locations of $X_{h}^{*}$ and $X_{c}^{*}$ depend on the strength of the sink function’s coefficient β(*N*).

We define then the following supersaturation values $\lambda_{h}=f\left(X_{h}^{*}\right)-g\left(X_{h}^{*}\right)$, corresponding to the local maximum of *f-g*, and $\lambda_{c}=f\left(X_{c}^{*}\right)-g\left(X_{c}^{*}\right)$, corresponding to its local minimum. In particular, λ_h_ ≥ λ_c_.

**Fig. 2.**
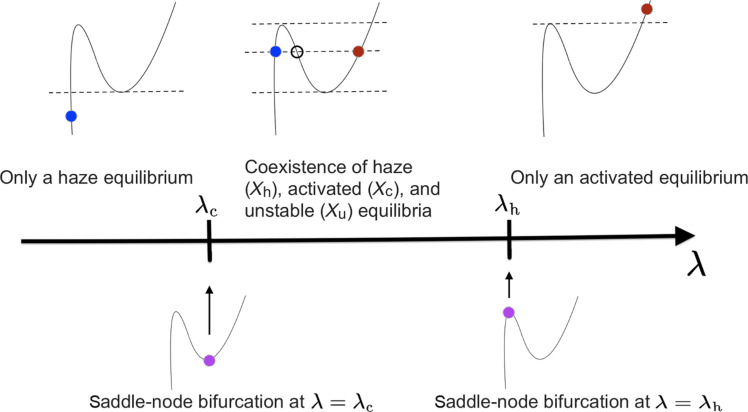
Going through multiple equilibria as λ varies. Two SN bifurcations occur, one at λ = λ_c_ and one λ = λ_h_. For λ < λ_c_, there is only a haze stable equilibrium *X*_h_ (blue dot in the leftmost upper diagram). As one crosses from below the SN bifurcation point at λ = λ_c_, two other equilibria emerge for a total of three equilibria coexisting for λ_c_ < λ < λ_h_ (middle upper diagram): an activated stable equilibrium *X*_c_ (red dot), an unstable equilibrium *X*_u_ (open circle), and a haze equilibrium (blue dot). At the SN bifurcation occurring at λ = λ_h_, *X*_u_ and *X*_h_ collide (purple dot on the rightmost lower diagram), and only the activated equilibrium survives as λ > λ_h_ (red dot in the rightmost upper diagram).

Because of the introduction of a new local minimum compared to the original Köhler curve (Fig. 1A), the number of equilibria varies with changes in supersaturation, λ, through two SN bifurcations. As depicted in Fig. 2, these bifurcations occur when the haze equilibrium (solid blue dot) approaches and merges with the unstable node (empty circle). Conversely, as λ decreases, a similar bifurcation takes place when the activated equilibrium approaches the unstable node. The solid and purple dots in the bottom panels denote the critical values of λ at which these bifurcations occur. These two SN bifurcations serve as the nonlinear foundation for the hysteresis phenomenon discussed in the “Hysteresis effects” section. These hysteresis effects are well known to be revealed in the presence of stochastic disturbances combined with slow parameter variations [see, e.g., (*39*)]. We discuss below the physical motivation of introducing such disturbances in Eq. 6 or, more generally, in Eq. 3.

### Brownian models and turbulent effects

In a developing cloud, turbulent updrafts and mixing with its neighboring air provoke supersaturation fluctuations that naturally affect droplet growth and, consequently, the collective droplet size spectra. In that respect, we refer to (*40*, *41*) for observational studies in stratocumulus clouds, and to (*42*, *43*) regarding the influence of temperature and pressure fluctuations in supersaturation distribution. Stochastic models for the velocity fluctuations and the supersaturation fields have been considered in previous studies and have shown various degrees of relevance (*23*, *37*). For a cloud parcel subject to turbulent updrafts, the authors in (*23*) prove that activated droplet size variance increases, at short times, proportionally to the square root of time, analogous to Einstein’s diffusion formula. Their analytical findings are contrasted with direct numerical simulations of turbulence obeying the incompressible Navier-Stokes equation. Despite this diffusion result with diverging variance, in (*37*), the authors find a statistical steady state for droplet size, with DSDs characterized by exponential tails (see their equation 4.3). Noteworthy is that to achieve convergence to a steady state, a substantial proportion of the droplets must reach evaporation, possibly if, on average, they experience negative supersaturations.

Supersaturation being the driving parameter, we assume that it evolves around a given value λ with fluctuations encoded by a stochastic term possibly depending on the particle size. The rationale behind this modeling assumption is that larger droplets are expected to have a greater impact on the supersaturation budget than the smaller ones, in a limited water vapor field. This is what we here referred to as inhomogeneous fluctuations, the inhomogeneous character being dependent on the droplets’ size. The type of inhomogeneity referred to here should not be confused thus with that caused by inhomogeneous mixing in clouds, occurring under large entrainment events (*44*).

These considerations invite us to parameterize the fluctuations of λ by means of a size-dependent stochastic term, $\sigma \left(X\right){\overset{\cdot }{W}}_{t}$, leading to the following stochastic model

$$\overset{\cdot }{X}=\underset{-V_{\lambda }^{\prime }\left(X\right)}{\underbrace{\lambda -f\left(X\right)+g\left(X\right)}}+\sigma \left(X\right){\overset{\cdot }{W}}_{t}$$ (8)

where *W*_t_ is a Brownian motion (and ${\overset{\cdot }{W}}_{t}$ is Gaussian and white). An example of the σ(*X*) function is given in the “Pi-chamber empirical distributions versus Gibbs states” section.

The function *V*_λ_ is the potential function that collects the nonlinear deterministic effects in the model. Thus, the solutions *X*(*t*) to Eq. 8 can be regarded as Brownian particles embedded in the potential *V*_λ_ defined in Eq. 8. The equilibria of the deterministic counterpart of Eq. 8 (i.e., Eq. 3) correspond exactly to the local critical points of the potential function—local minima in Fig. 1C—and their stability is determined by the local curvature of *V*_λ_ at those points [see, e.g., (*39*, *45*)]. Note that Eq. 8 can always be recast into a form in which the stochastic disturbance term becomes *X* independent, at the expense of changing the potential function via the Lamperti transformation [see chapter 3.6 in (*45*)]. This trick is recalled in the “The Lamperti transformation” section in Materials and Methods as it is also part of our model’s analysis below.

For a family of monodisperse droplets in the same cloud volume, each one experiences different supersaturation fluctuations, albeit with the same mean and variance, as encapsulated by the term $\sigma \left(X\right){\overset{\cdot }{W}}_{t}$ in Eq. 8. When each supersaturation’s stochastic realization is averaged over a large number of particles, the Fokker-Planck equation (FPE) associated with Eq. 8 provides the DSD at a given time *t* [see, e.g., (*45*, *46*)]. The stationary solution to the FPE is then given analytically and known as the Gibbs state [see (*33*) and equation 4.35 in (*45*)]

$$\rho_{\lambda }\left(X\right)=Z_{\lambda }^{-1}\frac{\text{exp}\left[2\int^{X}\frac{\lambda -f\left(x\right)+g\left(x\right)}{\sigma^{2}\left(x\right)}\;dx\right]}{\sigma^{2}\left(X\right)}$$ (9)

where *Z*_λ_ is a normalizing constant. This distribution is a generalization of Weibull distribution found in equation 6 in (*47*) to explain diffusive broadening of the DSDs in turbulent clouds. Within our framework, it corresponds to Gibbs states without sink function and state dependency in the noise, i.e., density distribution ρ_W_ (for the radius *r*) obtained by setting *f* = *g* ≡ 0 and σ constant. In this particular case, the resulting DSD is integrable only if there is mean subsaturation, λ < 0, and is given by

$$\rho_{W}\left(r\right)=Z_{\lambda }^{-1}{\mathrm{re}}^{\gamma r^{2}}$$ (10)

where γ is a parameter proportional to λ, droplet number concentration, and liquid water fraction [see equation 8 in (*47*)]. In comparison, ρ_λ_ in Eq. 9 can be integrable if λ is positive and subject to size-dependent fluctuations, provided that the sink function *g* has suitable decay properties such as detailed in Proposition 2 (see the “The confining potential” section in Materials and Methods). We refer to (*32*) for other theoretical approaches to infer integrable DSDs for fluctuating supersaturation with size-dependent removal terms.

Our framework, for a broad class of functions *f*, *g*, and σ, allows for Gibbs state ρ_λ_ that exhibits multimodality intimately related to shape of the potential *V*_λ_. More precisely, the Gibbs state’s modes, the local maxima of ρ_λ_, are found by solving the following equation

$$-V_{\lambda }^{\prime }\left(X\right)=\frac{1}{2}\sigma \prime \left(X\right)\sigma \left(X\right)$$ (11)

See the “Noise-induced metastability” section in Materials and Methods for the derivation of this equation. This fundamental equation teaches us how the Gibbs state modes result from the interaction between the noise term and the potential function. The Gibbs state’s modes correspond to metastable states for which the random fluctuation term, $\sigma \left(X\right){\overset{\cdot }{W}}_{t}$, in Eq. 8 acts as a source for the particles to experience transitions from a statistically typical droplet size to another one.

When the noise is state independent, i.e., when $\sigma \left(X\right)=\sqrt{2\varepsilon }$, for some positive constant ε > 0 (controlling the noise variance), then σ′(*X*) = 0 and the metastable states are directly determined by the zeros of −*V*_λ_′. In this case, for Eq. 6, the Gibbs states are bimodal when λ_c_ < λ < λ_h_, and unimodal when there is just one underlying stable equilibrium, i.e., for either λ < λ_c_ or λ > λ_h_, as shown in Fig. 2, whereby the solid points denote the presence of equilibria, ranging from a single haze equilibrium to an activated one.

**Fig. 3.**
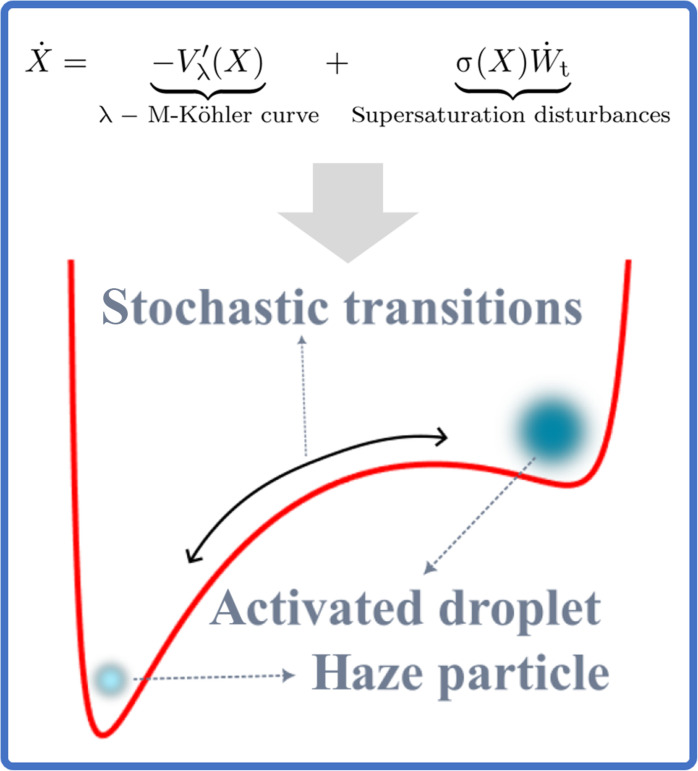
Cartoon of haze-to-droplet stochastic transitions. Here, λ is kept fixed and the situation depicted corresponds to λ in (λ_c_, λ_h_), where two stable states coexist (haze particle and activated droplet) (see Fig. 2).

When λ_c_ < λ < λ_h_, there is a notable chance of encountering haze or activated droplets. As depicted in Fig. 3, the red curve represents a potential energy landscape with two local minima similar to that shown in Fig. 1C. Stochastic fluctuations can then overcome the potential barrier between these two minima, leading to activation even when average supersaturation falls below the Köhler threshold. This phenomenon aligns with real-world scenarios where turbulent supersaturation variations can induce haze activation (*28*).

Our modeling framework allows for other useful analytic insights. For instance, the expected residence times at the haze or activated state can be precisely determined. They are indeed known to relate to the potential barrier, *V*_λ_(*X*_u_)−*V*_λ_(*X*_h_) (see Fig. 1C), according to the Kramers’ time formulas (*39*, *48*). The mean activation time τ_act_ is then given by equation 2.19 in (*49*)

$$\tau_{\text{act}}\left(\lambda \right)=\frac{2\pi }{\sqrt{|V_{\lambda }^{\prime \prime }\left(X_{u}\right)|V_{\lambda }^{\prime \prime }\left(X_{h}\right)}}e^{\frac{V_{\lambda }\left(X_{u}\right)-V_{\lambda }\left(X_{h}\right)}{\varepsilon }}$$ (12)

Therefore, the closer λ is to the critical value λ_h_ from below, the closer *X*_u_ is to *X*_h_ (see Fig. 2) and the shallower the potential barrier becomes, resulting into more common particle activation. Similarly, deactivation of activated droplets can take place due to fluctuations in supersaturation with a mean deactivation time obtained by Eq. 12 in which *X*_h_ is replaced by *X*_c_.

### Hysteresis effects

The hysteresis behavior pointed out at the end of the “Multistable Köhler curves” section is now analyzed when the supersaturation parameter λ is allowed to drift through the critical values λ_c_ and λ_h_, slower compared to the stochastic fluctuations. Such relatively slower variations in λ are associated with buoyancy-driven supersaturation changes. Supersaturation λ increases as the buoyant parcel rises until the activation threshold λ_h_ is attained and some of the contained haze is activated. When the parcel ceases to be buoyant or reaches a temperature inversion, supersaturation decreases to λ_c_, below which the whole family of droplets regenerates into haze.

**Fig. 4.**
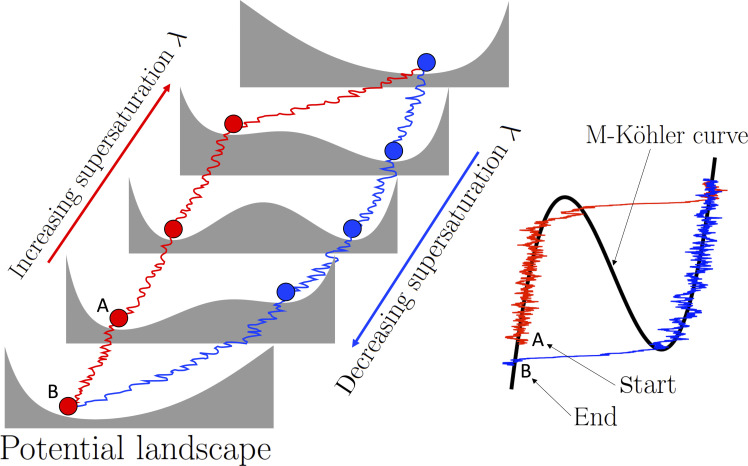
Rationale of a hysteresis path. As λ varies, the potential landscape changes and the stochastic disturbances can help trigger the critical transition as one approaches the tipping points, here the SN bifurcation points.

During this buoyancy cycle, the population of particles faces a continuous change in the potential *V*_λ_, as illustrated in Fig. 4, for increasing and decreasing supersaturation levels. It is there shown the asymmetry of activation. When a haze particle undergoes activation—starting at point A and following the red wobbly line—its deactivation will occur at much lower supersaturation levels, as seen when following the blue wobbly line that ends in point B. Similar cycles have also been reported in the absence of aerosol curvature and chemistry effects, and uniquely due to noisy supersaturation fluctuations around a subsaturated mean [see, e.g., figure 4 in (*37*)].

Within the present framework, it remains to investigate the effects of (i) different rates of changes in supersaturation and (ii) variations in the magnitude of the fast supersaturation fluctuations as encapsulated by the noise term. The hysteresis phenomenon under the presence of noise has been investigated in previous work (*50*), where it is found that a large (additive) noise variance σ, on average, reduces the area of hysteresis loops. In our context, this implies that droplet activation can occur at much lower supersaturation values compared to λ_h_. To numerically examine these ideas, we solve Eq. 8 associated with the sink term *g*(*X*) = −β(*N*)*X*^3/2^ used in Eq. 6, for specific noise variances and mean supersaturation λ ranging from 0.06% to 0.11% with different rate of change $\overset{\cdot }{\lambda }$. We refer to the “Hysteresis path algorithm” section in Materials and Methods for numerical details.

**Fig. 5.**
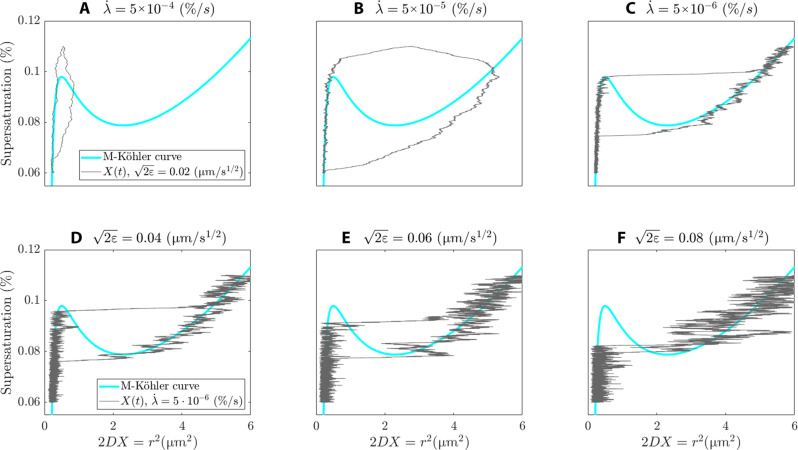
A landscape of hysteresis loops. (**A** to **C**) The thin gray line indicates the hysteresis loops for different rates of change of supersaturation $\overset{\cdot }{\lambda }$ as indicated in the title. The noise variance is constant for these three panels as indicated in the legend of (A). (**D** to **F**) The thin gray lines indicate the hysteresis loops for different noise variances as indicated in the title. The rate of change of supersaturation $\overset{\cdot }{\lambda }$ is constant for these three panels as indicated in the legend of (D). For all panels, the cyan line is the M-Köhler curve associated with Eq. 6 with β(*N*) = 3.6 × 10^−2^ s^−3/2^.

Our model supports different hysteresis cycles as shown in Fig. 4. The nature of these hysteresis loops is controlled by the rate of change of supersaturation $\overset{\cdot }{\lambda }$ and the intensity of the turbulent effects. Figure 5 (A to C) corresponds to an increasingly faster response of droplet growth to supersaturation changes. Figure 5 (D to F) corresponds to an increasing susceptibility to activation as the noise variance is increased, i.e., the turbulent effects are more pronounced, while the rate of change $\overset{\cdot }{\lambda }$ is kept fixed.

As expected, when the rate of change in supersaturation is not slow enough, it can prevent droplet activation (see Fig. 5A), whereas sufficiently slow adiabatic changes enforce the droplet size to follow the M-Köhler curve such as shown in Fig. 5C. As a haze particle experiences an increase in supersaturation, resulting from an adiabatic cooling of a cloud parcel, this will grow by condensation following the Köhler curve, almost regardless of how intense the cooling rate is, here indicated by $\overset{\cdot }{\lambda }$. This is an indication of the stability of the thermodynamic equilibrium of haze particles as opposed to cloud droplets, which are more sensitive to the cooling rate $\overset{\cdot }{\lambda }$. By comparing Fig. 5B and C, we observe that only when the cooling rate is slowest do we observe a droplet growth attached to the Köhler curve.

Figure 5 (D to F) provides for Eq. 6 illustrations of hysteresis cycles that are consistent with those analyzed for large-noise regimes in theoretical studies (*50*). Compared to Fig. 5 (A and B), the rate of change of supersaturation $\overset{\cdot }{\lambda }$ is smaller, resulting into sharper transitions to activation similar than those shown in Fig. 5C. This, combined with a larger turbulent noise intensity than in the cases of Fig. 5 (A to C), may provoke transitions to occur before reaching the haze-to-droplet SN bifurcation, i.e., particles that activate at much lower supersaturations compared to that predicted by Köhler. Physically, it corresponds to haze particles that grow in a slowly cooling air volume embedded in a highly turbulent environment undergoing heaving turbulent updrafts. In some marked limit, the hysteresis loop may collapse such as shown in Fig. 5F. This extreme situation is to put in contrast with the other extreme situation shown in Fig. 5A, where activation is “unrealized” due to the faster rate of change of supersaturation and weakly turbulent updraft.

### Pi-chamber empirical distributions versus Gibbs states

Small warm convective clouds are abundant in the atmosphere but hard to measure (*51*). Such clouds are often characterized by weak updrafts and small supersaturation, allowing for a substantial part of the aerosol to coexist as haze together with the activated part (*34*, *35*). Cloud chambers are often used to study clouds in such thermodynamic conditions experimentally (*28*, *52*).

In such work, the so-called Pi-chamber is seeded with monodisperse families of particles, and turbulent fluctuations are induced by changes in temperature, pressure, and aerosol injection rates. Disregarding transients, the system reaches a steady state in number concentration and DSDs are calculated spanning haze and active particles. Depending on the supersaturation mean and variance, DSDs display different characteristics involving their multimodality and spread [see figure 2 in (*28*)]. We show here that such experimental distributions are well approximated by the Gibbs states of our Brownian modeling framework once the sink and noise terms are appropriately designed. Our goal is to provide a qualitative and quantitative correspondence between the present theory and the experiments from (*28*).

We first address the sink term design. Note that for a monodisperse family of particles embedded in a cloud parcel, the supersaturation budget must be included in the collective droplet growth (*10*, *53*). Because the Pi-chamber is in steady state, the sink term affecting supersaturation is determined by water vapor condensation onto the particles, which boils down to assuming that supersaturation is consumed proportionally to the radius of the droplets. Thus, a sink term of type *g*(*X*) = −β*X*^1/2^ is chosen in Eq. 8, where the absorption parameter β depends on the number of active droplets and the effective diffusivity of water vapor in air (*6*). However, it must be noted that, although in steady state, the Pi-chamber is not a closed system. There is an intrinsic decorrelation time scale of supersaturation [see (*52*)]. This time scale is usually determined by the coefficients in front of the deterministic terms of a (linear) stochastic Langevin equation governing the supersaturation evolution [see, e.g., equation 2 in (*52*)].

In our formalism, while not solving an explicit governing equation for supersaturation, we can still determine a decorrelation time scale for supersaturation, albeit in an indirect fashion. One first solves Eq. 8, records *X*(*t*), and then forms supersaturation time series according to

$$\lambda \left(t\right)=\lambda -\beta X{\left(t\right)}^{1/2}+\sigma \left[X\left(t\right)\right]{\overset{\cdot }{W}}_{t}$$ (13)

Now, the expectation of the stochastic term $\sigma \left(X\right){\overset{\cdot }{W}}_{t}$ is zero due to the martingale property of Brownian motion and the independence of σ[*X*(*t*)] and ${\overset{\cdot }{W}}_{t}$. This is actually a fundamental property of stochastic differential equations (SDEs) driven by Brownian motion [see section 4.2.6 in (*54*)]. Therefore, the expected value of supersaturation has a decorrelation time given by that of $\lambda -\beta EX{\left(t\right)}^{1/2}$, that is, a function of the decorrelation time of the droplet radius. Thus, the droplet size effects on supersaturation fluctuations encoded by the stochastic term $\sigma \left(X\right){\overset{\cdot }{W}}_{t}$ affect the decorrelation time of the expected value of supersaturation in an indirect way, after solving Eq. 8 to get access to *X*(*t*).

In the case of the Pi-chamber datasets analyzed below, we model the size-dependent effects on supersaturation fluctuations via the following functional

$$\begin{array}{c} \sigma \left(X\right)=\left\{\sigma_{1}+\frac{\sigma_{2}-\sigma_{1}}{2}\left[1+\psi \left(X\right)\right]\right\}\\ \psi \left(X\right)=\text{tanh}\left[2\kappa D\left(X-X^{*}\right)\right] \end{array}$$ (14)

where we require that σ_2_ > σ_1_ > 0 so that larger droplets have a bigger impact on supersaturation fluctuations than the smaller ones. The value of the slope is chosen to be sufficiently large (i.e., 2κ*D* = 10) so that the hyperbolic tangent function, ψ, approximates a step function while still exhibiting the attributes of a smooth function, more amenable for analysis and numerical simulations. The “ignition” parameter *X*^∗^ is chosen to be equal to 6.2 × 10^−3^ s or, in diameter, *d*^∗^ = 1.41 μm, which yields a correct location of the haze mode, and it is in the order of the critical Köhler diameter, which is *d*_K_ = 1.7 μm. Such a critical value is derived from Eq. 1, as explained in Köhler theory, for the curvature and solubility coefficients *A* = 1.4 × 10^−3^ μm and *B* = 3.5 × 10^−4^ μm^3^ [see (*28*)].

We thus arrive at the following Brownian model to describe cloud droplet size evolution in the Pi-chamber

$$\overset{\cdot }{X}=\lambda -f\left(X\right)-\beta X^{1/2}+\sigma \left(X\right){\overset{\cdot }{W}}_{t}$$ (15)

with σ given by Eq. 14, and we recall that *f* is given by Eq. 2.

By solving Eq. 11 associated with Eq. 15, we can derive a relationship (valid for *X* sufficiently bigger than *X*^∗^) determining the parameter β in terms of the modal size *X*_c_ of the activated mode

$$\beta =\frac{1}{X_{c}^{2}}\left[\lambda X_{c}^{3/2}-\frac{A}{{\left(2D\right)}^{1/2}}X_{c}+\frac{B}{{\left(2D\right)}^{3/2}}\right]$$ (16)

Equation 16 plays a key role to estimate β: Once β is known, the only parameters left for calibration of the model are σ_1_, σ_2_, and the stiffness parameter κ.

We now confront the ability of our Brownian model (Eq. 15) to approximate experimental DSDs by the Gibbs states formula (Eq. 9). Three experimental setups are considered in that respect, following those of the Pi-chamber experiments from (*28*) corresponding to different supersaturation mean and fluctuation properties reported in such paper. These are referred to as according to (*28*): (i) mean-dominated, (ii) fluctuation-influenced, and (iii) fluctuation-dominated activation.

Within our modeling framework, these regimes can be organized as follows:

Case I: λ > λ_K_ → mean-dominated activation.

Case II: λ_c_ < λ < λ_K_ → fluctuation-influenced activation.

Case III: λ < 1 → fluctuation-dominated activation.

The parameter λ_K_ is the critical Köhler threshold, which is derived to be λ ≈ 1.00108, for the type of aerosols considered in (*28*). The steady-state supersaturation value in each case is also derived from (*28*). Their theoretical calculations reveal that case I corresponds to λ = 1%. While the value for case II is not explicitly provided, it can be inferred from our framework that λ is close to λ_h_ from below since a bimodal DSD is observed, with high peaks in the haze and activated domain: λ = 0.1% is taken for this case. Finally, case III is a subsaturated regime (λ < 0), and as such, we choose λ to be given by the Köhler’s value *f*(*X*) at the haze peak’s location *X* = *X*_h_; this gives λ = −1%.

**Fig. 6.**
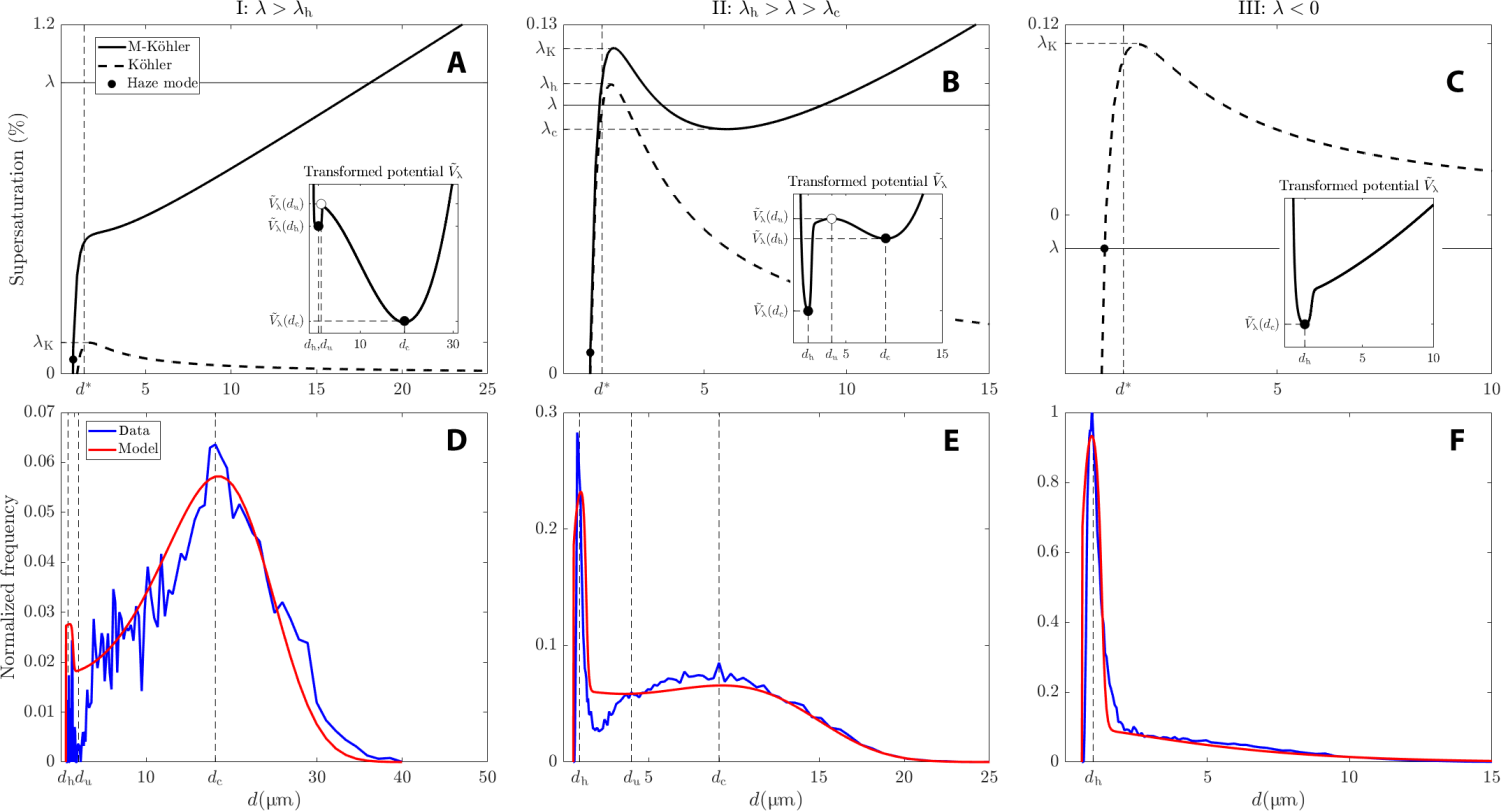
Pi-chamber experimental distributions versus Gibbs states, and underlying M-Köhler curves. (**A** to **C**) Köhler and M-Köhler curves (dashed and solid curves, respectively) for each supersaturation value considered in the “Pi-chamber empirical distributions versus Gibbs states” section (cases I, II, and III). The parameters of the Köhler curve are *A* = 1.4 × 10^−3^ μm and *B* = 3.5 × 10^−4^ μm^3^, and the other parameters of model Eq. 15 are given in Table 2. The black dot denotes the location of the metastable state induced by the state-dependent noise (see the “Noise-induced metastability” section in Materials and Methods). (**D** to **F**) Experimental DSDs from (*28*) versus Gibbs states from Eq. 9. Both the data histograms and Gibbs states are normalized according to their integral. The insets show the transformed potentials (due to Lamperti transformation) explaining the shapes of the Gibbs states (see the “The Lamperti transformation” section in Materials and Methods).

With these parameter values for λ, the parameter β can be estimated for cases I and II according to Eq. 16, in which *X*_c_ is chosen to be the experimental DSD peak’s location that corresponds to activated droplets. For case I, the modal activated diameter is *d*_c_ = 18.109 μm, giving *X*_c_ = 1.025 s and β = 9.7 × 10^−3^ s^−1/2^. In case II, the modal activated diameter is *d*_c_ = 9.141 μm, giving *X*_c_ = 0.2611 s and β = 1.4 × 10^−3^ s^−1/2^. These modal values are obtained from the data of (*28*). For case III, though, since the system is subsaturated and haze particles are more numerous [see table 1 of (*28*)], the term β is practically zero. The corresponding original Köhler and M-Köhler curves are shown in Fig. 6 (A and B), as dashed and solid curves, respectively, and shown as functions of the diameter $d=2\sqrt{2\mathrm{DX}}$. For case III, because there is no sink term, only the original Köhler curve is shown in Fig. 6C.

Once the parameters for the relative disturbances σ_1_, σ_2_, and κ are chosen as listed in Table 2, the corresponding Gibbs states ρ_λ_ given by Eq. 9 exhibit striking skills in reproducing the empirical DSDs in each of the three Pi-chamber experiments taken from (*28*). The results are shown in Fig. 6D to F. The shapes of the Gibbs states are highly correlated to those of the transformed potentials shown in the insets of Fig. 6, obtained by the Lamperti transformation (see the “The Lamperti transformation” section in Materials and Methods). We observe in particular that the potential wells’ locations match the Gibbs states modes’ locations. Recall that the latter transformation allows for identifying the effective potential for the stochastic model (Eq. 15) subject to size-dependent stochastic disturbances such as given by Eq. 14. With this effective potential, Eq. 15 can be interpreted as a Brownian particle evolving within that potential whose fluctuations are just driven by a white noise, independently of the size of the particle.

**Table 2.** Supersaturation values, sink term’s parameters, and state-dependent noise’s parameters.

	λ × 100 (%)	β (s^−1/2^)	σ_1_ (s^1/2^)	σ_2_ (s^1/2^)	d* (μm)	2κD
Case I	1	9.6 × 10^−3^	3.75 × 10^−2^	6.25 × 10^−2^	1.41	10
Case II	0.1	1.4 × 10^−3^	7.5 × 10^−3^	1.5 × 10^−2^	1.41	10
Case III	−1	0	5 × 10^−3^	1.5 × 10^−2^	1.41	10

**Fig. 7.**
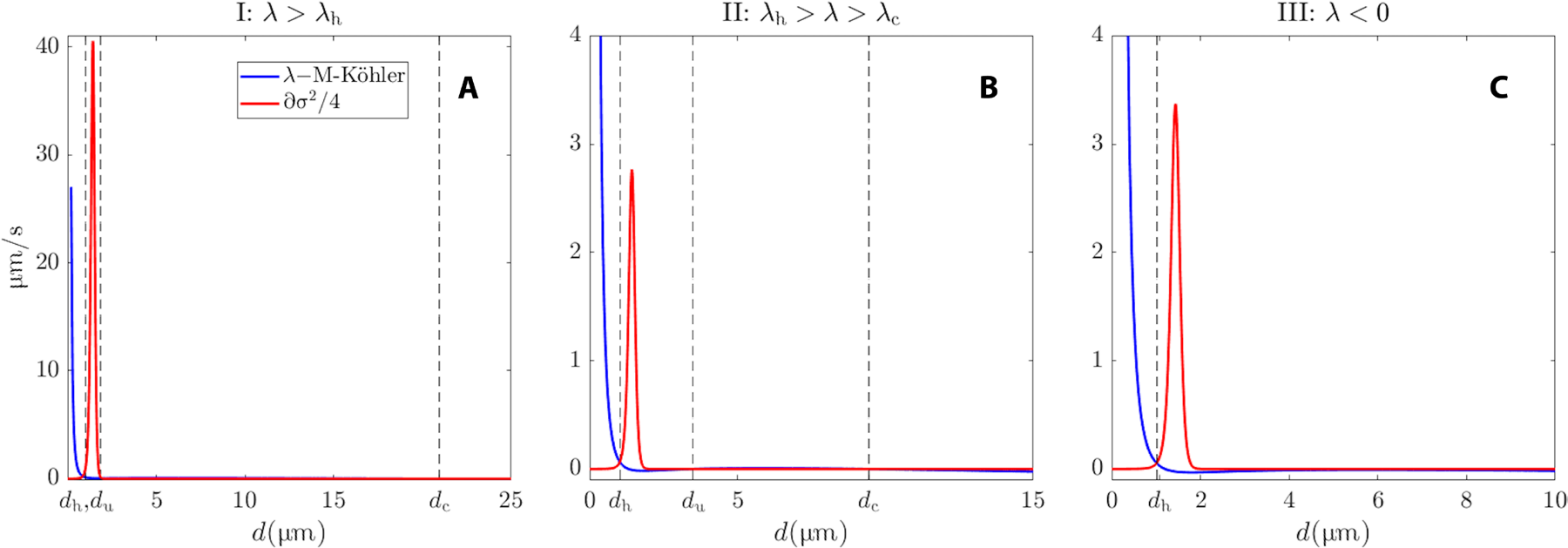
Noise-induced metastable states. (**A** to **C**) Three different supersaturation configurations as indicated in the titles and corresponding to those of Fig. 6. The blue curves show the growth rate function −*V*′_λ_ in Eq. 8, such as associated with the M-Köhler curve *f*(*X*) + β*X*^1/2^. The red curves show the derivative of the noise covariance after division by a factor of 4 corresponding to the right-hand side of Eq. 34. The vertical dashed lines indicate the location of the solutions to Eq. 34, which also correspond to the haze, unstable, and activated metastable states, denoted by *d*_h_, *d*_u_, and *d*_c_, respectively.

The theory allows us to make striking predictions. For instance, the experimental DSDs’ modes are well predicted by Eq. 11, as shown in Fig. 7 of the “Noise-induced metastability” section in Materials and Methods, especially in cases I and II, where the classical Köhler theory is unable to predict an activated mode, since λ > λ_K_. Noteworthy is the creation of a metastable haze state in case I resulting from the interaction between the noise term $\sigma \left(X\right){\overset{\cdot }{W}}_{t}$ and the nonlinear drift terms −*f*(*X*) − β*X*^1/2^ as revealed by the transformed potential shown in the inset of Fig. 6D (see the “Noise-induced metastability” section in Materials and Methods).

## DISCUSSION

Köhler theory is at the basis of cloud physics. For a given dry aerosol size and composition, it predicts the particle’s state, haze, or activated droplet as a function of the particle size and the supersaturation. The Köhler curve (Fig. 1A) has two fixed-point regimes bounded by the curve’s mode. The curve left to the mode describes a stable fixed point, whereas any particle that resides on the curve right to the mode is on an unstable fixed point such that a perturbation to the right (or upward) will shift the particle to a continuous growth by diffusion and a perturbation to the left (or downward) will send the particle to the haze state. Warm and small clouds that are driven by weak temperature or humidity perturbations (*5*, *34*, *35*) are abundant and have an important radiative effect. However, due to their sizes, weak optical signature, and short lifetimes, these clouds are mostly overlooked, and their properties are poorly understood. Such clouds often exist near the transition between haze to cloud (*55*)—a regime that can be studied in cloud chambers (*28*, *52*).

Here, we address these clouds by extending Köhler theory to a population of droplets embedded in turbulent environments. While activation is explained by Köhler’s theory, the reverse process, i.e., deactivation, is dynamically different. It has been documented in previous works (*10*, *11*) and in the present study that deactivation is intimately tied to the hysteresis phenomenon through which deactivation occurs at a smaller supersaturation compared to the critical Köhler value λ_K_. The addition of a sink term in the condensational growth equation (see Eq. 3) yields hysteresis directly from an analytical perspective: To deactivate a family of monodisperse cloud droplets, the supersaturation needs to be brought strictly below the critical value λ_K_ predicted by Köhler. The origin of this behavior is the presence of another SN bifurcation that results from the bending of the M-Köhler curve introduced in the “Multistable Köhler curves” section. This bending translates further into the appearance of another stable equilibrium on the activated droplet spectrum as a balance between condensational instability and sink factors, like supersaturation consumption or particle removal.

The introduction of Brownian noise in the condensational growth equation parameterizes the influence of supersaturation fluctuations and gives analytical formulas for computing DSDs, here identified as Gibbs states (see Eq. 9). Such probability distributions generalize earlier formulas for DSDs found in the literature such as Weibull distributions (*31*, *32*, *47*).

The resulting DSDs are categorized into three types based on the mean supersaturation parameter λ. In the first type, fluctuations in supersaturation are insufficient to sustain droplet activation for extended periods, leading to a unimodal DSD centered around the haze state. In the second type, activation is possible, and when the threshold λ_h_ is exceeded, haze particles become unstable. This results in a single DSD mode peaking at activated sizes. Notably, even if Köhler’s threshold supersaturation is not reached, there is a finite probability of particle activation. The associated activation timescales are linked to the average time required for droplets to overcome the activation energy barrier (Fig. 1C), as accurately described by Kramers’ formula (Eq. 12).

When a cloud parcel undergoes adiabatic cooling, supersaturation fluctuates around a gradually increasing mean. Ignoring turbulent fluctuations can lead to an overestimation of the activation threshold, as illustrated in Fig. 5. This figure depicts various hysteresis loops where activation occurs at even lower supersaturation values than λ_h_, especially when the noise variance is large.

Our stochastic M-Köhler model has demonstrated remarkable agreement with steady-state cloud experiments conducted in the Pi-chamber. Notably, it accurately captures the intricate DSD shapes, including bimodal features, as observed in the experimental study by (*28*) across various saturation regimes. To further explore this consistency, future investigations should focus on slow adiabatic changes in mean supersaturation. On the basis of the analytical understanding of droplet activation-deactivation hysteresis (Fig. 5), we anticipate that similar hysteresis behavior will be observed in the experimental setup of (*28*).

In natural environments, weak clouds often encounter aerosols of varying sizes and compositions. Nevertheless, the low supersaturation levels may act as a selective filter, favoring the activation of larger haze particles. This can create conditions that are not substantially different from our theoretical framework. Additionally, the presence of Brownian motion can introduce variability into the particle distribution, even with initially monodisperse conditions. Future studies will explore scenarios involving polydisperse particles with diverse chemical properties and varying Köhler curves.

## MATERIALS AND METHODS

### Multistability

To ensure that the model defined by Eq. 3 exhibits multiple stable equilibria, it suffices for the M-Köhler curve *f-g* to have at least one local minimum and one local maximum. This condition guarantees that the roots of the equation λ − (*f* − *g*) = 0 correspond to at least two stable equilibria and one unstable equilibrium within a certain range of λ values.

To simplify the presentation of the results stated in Proposition 1, we introduce the following parameters

$$\tilde{A}=\frac{A}{{\left(2D\right)}^{1/2}}$$ (17a)

$$\tilde{B}=\frac{B}{{\left(2D\right)}^{3/2}}$$ (17b)

We then have the following sufficiency conditions for the existence of a local maximum and a local minimum:

**Proposition 1** Let *f* be the function given in Eq. 2 and let *g* : ℝ^+^ → ℝ^−^ be a function of the form *g*(*X*) = −β*X*^α^, where β > 0 and α > 1. If $\overset{\cdot }{g}\left(5\tilde{B}/\tilde{A}\right)>\overset{\cdot }{f}\left(5\tilde{B}/\tilde{A}\right)$, the M-Köhler curve in Eq. 3 has at least a local maximum and a local minimum.

*Proof*. To demonstrate the existence of two zeros for the function $\overset{\cdot }{f}-\overset{\cdot }{g}$, it is sufficient to prove that it undergoes at least two sign changes. For *X* > 0, this function is given by

$$\overset{\cdot }{f}\left(X\right)-\overset{\cdot }{g}\left(X\right)=-\frac{\tilde{A}}{2}X^{-3/2}+\frac{3\tilde{B}}{2}X^{-5/2}+\alpha \beta X^{\alpha -1}$$ (18)

When α = 3/2 as in Eq. 6, one recovers that finding the zeros of $\overset{\cdot }{f}-\overset{\cdot }{g}$ is equivalent to finding the roots of the polynomial Eq. 7.

First, $\overset{\cdot }{f}$ has a single local and global minimum. This can be observed by examining $\ddot{f}$

$$\ddot{f}\left(X\right)=\frac{3}{4}\tilde{A}X^{-5/2}-\frac{15}{4}\tilde{B}X^{-7/2}$$ (19)

which has a unique zero at $X=5\tilde{B}/\tilde{A}$, and

$$\overset{\cdot }{f}\left(\frac{5\tilde{B}}{\tilde{A}}\right)=-\frac{\tilde{A}}{5}{\left(\frac{\tilde{A}}{5\tilde{B}}\right)}^{\frac{3}{2}}<0$$ (20)

Furthermore, the two limits below hold

$$\underset{X\to 0^{+}}{\text{lim}}\overset{\cdot }{f}\left(X\right)=+\infty$$ (21a)

$$\underset{X\to \infty }{\text{lim}}\overset{\cdot }{f}\left(X\right)=0$$ (21b)

These two limits together with Eq. 20 indicate that $X=5\tilde{B}/\tilde{A}$ is a global minimum.

Because of the smoothness of *f* and *g* on ℝ^+^, the limit in Eq. 21a, and—by hypothesis—α > 1, we have

$$\underset{X\to 0^{+}}{\text{lim}}\overset{\cdot }{f}\left(X\right)-\overset{\cdot }{g}\left(X\right)=+\infty$$ (22)

there exists *X*_1_ > 0 such that $\overset{\cdot }{f}\left(X_{1}\right)-\overset{\cdot }{g}\left(X_{1}\right)>0$. Moreover, by hypothesis, $\overset{\cdot }{f}\left(5\tilde{B}/\tilde{A}\right)-\overset{\cdot }{g}\left(5\tilde{B}/\tilde{A}\right)<0$, and that gives the first change of sign of the function $\overset{\cdot }{f}-\overset{\cdot }{g}$.

Last, by assumption, we have that α > 1, so the following limit holds

$$\underset{X\to \infty }{\text{lim}}\overset{\cdot }{g}\left(X\right)=\underset{X\to \infty }{\text{lim}}-\alpha \beta X^{\alpha -1}=-\infty$$ (23)

Considering this limit and the one in Eq. 21b, there exists $X_{2}>5\tilde{B}/\tilde{A}$ such that $\overset{\cdot }{f}\left(X_{2}\right)-\overset{\cdot }{g}\left(X_{2}\right)>0$. This gives the second change of sign.

Because of the continuity and smoothness of the functions *f* and *g* on R^+^, the function $\overset{\cdot }{f}-\overset{\cdot }{g}$ must have at least two zeros.

### The confining potential

In order for a gradient system to have a Gibbs state, it is necessary that the associated potential is confining [see, e.g., chapter 4 in (*45*)]. We provide here sufficient mathematical conditions by which a sink function *g* in Eq. 8 ensures the confining property. Generally, it amounts to imposing a sufficiently strong decay rate to the sink term.

The potential function *V*_λ_ is said to be confining if

$$\underset{X\to 0^{+}}{\text{lim}}V_{\lambda }\left(X\right)=\underset{X\to \infty }{\text{lim}}V_{\lambda }\left(X\right)=\infty$$ (24)

and if for any ξ > 0

$$\int_{0}^{\infty }e^{-{\mathrm{\xi V}}_{\lambda }\left(X\right)}\;dX<\infty$$ (25)

Below, we provide a class of sink functions for which the associated M-Köhler curve has a confining potential.

**Proposition 2** Let λ be in ℝ, *f* be as in Eq. 2, and *g* : ℝ^+^ → ℝ^−^ be defined as *g*(*X*) =−β*X*^α^, for *X*, α, β > 0. Then, the potential *V*_λ_ associated with Eq. 8 is confining.

*Proof*. By the definition of *f*, *g*, and the potential *V*_λ_, we find that

$$-V_{\lambda }\left(X\right)=\int^{X}\left[\lambda -f\left(X\right)+g\left(X\right)\right]\;dX$$ (26a)

$$=\lambda X-2\tilde{A}X^{1/2}-2\tilde{B}X^{-1/2}-\frac{\beta }{1+\alpha }X^{1+\alpha }$$ (26b)

where the parameters $\tilde{A}$ and $\tilde{B}$ are defined in Eq. 17. Then

$$\underset{X\to 0^{+}}{\text{lim}}V_{\lambda }\left(X\right)=\underset{X\to 0^{+}}{\text{lim}}2\tilde{B}X^{-1/2}=\infty$$ (27a)

$$\underset{X\to \infty }{\text{lim}}V_{\lambda }\left(X\right)=\underset{X\to \infty }{\text{lim}}\frac{\beta }{1+\alpha }X^{1+\alpha }=\infty$$ (27b)

We are left with showing the integrability condition (Eq. 25). The integral of the exponential of the potential satisfies, for any ξ > 0, the following inequalities

$$\int_{0}^{\infty }e^{-\xi V_{\lambda }\left(X\right)}\;dX$$ (28a)

$$=\int_{0}^{\infty }e^{\xi \left(\lambda X-2\tilde{A}X^{1/2}-2\tilde{B}X^{-1/2}-\frac{\beta }{1+\alpha }X^{1+\alpha }\right)}\;dX$$ (28b)

$$\leq \int_{0}^{\infty }e^{\xi \left(\lambda X-\frac{\beta }{1+\alpha }X^{1+\alpha }\right)}\;dX$$ (28c)

$$=\int_{0}^{\infty }e^{-X\left(\frac{\xi \beta }{1+\alpha }X^{\alpha }-\xi \lambda \right)}\;dX$$ (28d)

$$\leq \int_{0}^{X_{0}}e^{-X\left(\frac{\xi \beta }{1+\alpha }X^{\alpha }-\xi \lambda \right)}\;dX+\int_{X_{0}}^{\infty }e^{-X}\;dX<\infty$$ (28e)

for any ξ > 0 and where *X*_0_ = [(1 + ξλ)(1 + α)/ξβ]^1/α^ is such that

$$\frac{\xi \beta }{1+\alpha }X_{0}^{\alpha }-\xi \lambda =1$$ (29)

This proves the confining property.

### Hysteresis path algorithm

To produce the hysteresis paths shown in Fig. 5, we solve Eq. 6 for slowly varying values of the supersaturation parameter λ and Brownian noise’s parameter σ taken to be additive, i.e., $\sigma \left(X\right)=\sqrt{2\varepsilon }$. The slow drift of the supersaturation parameter is organized as follows. We start by dividing an interval [λ_0_, λ_*N*+1_] ⊃ (λ_c_, λ_h_) uniformly into *N* + 2 grid points ${\left\{\lambda_{j}\right\}}_{j=0}^{N+1}$ such that λ*_j_* = λ_0_ + *j*δλ, with δλ = (λ_h_−λ_c_)/(*N*−1) and λ_0_ = λ*_c_*−δλ/2. Note that λ_*N* + 1_ > λ_h_ and λ*_N_* < λ_h_.

To form the lower branch of this hysteresis path, we start with λ = λ_0_ and from the haze state *X*_h_(λ_0_), and solve Eq. 6 using an Euler-Maruyama scheme with time-step δ*t* = 10^−2^ s [see chapter 9.1 in (*56*)].

Then, λ is updated to λ_0_ + *j*δλ and Eq. 6 is iterated over another time step by still using Euler-Maruyama. The process is repeated *N* + 1 times until λ_*N*+1_ > λ_h_. The reverse trajectory is obtained by repeating the algorithm, although starting with λ = λ_*N*+1_ at *X*_c_(λ_*N*+1_). More precisions are given in Algorithm 1 and hereafter.

**Figure:**
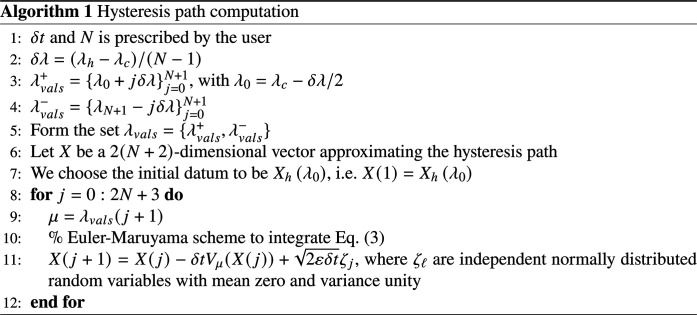


The key parameter to produce Fig. 5 (A to C) is $\overset{\cdot }{\lambda }$. Note that according to the previous algorithm, $\overset{\cdot }{\lambda }\approx \delta \lambda /\delta t$. Consequently, the larger *N* is, the smaller δλ, and vice versa.

### The Lamperti transformation

For scalar stochastic differential model, it is possible to map an SDE driven by state-dependent noise onto an SDE driven by an additive noise. We recall this basic idea. Consider the following scalar SDE

$$\overset{\cdot }{X}=-V\left(X\right)+\sigma \left(X\right){\overset{\cdot }{W}}_{t}$$ (30)

with *V* and σ smooth functions such that σ > 0. Consider the change of variables, $h\left(X\right)=\int^{X}\frac{1}{\sigma \left(X\right)}\;dX$, then by application of Itô’s formula, the original Eq. 30 is transformed into

$$\overset{\cdot }{Y}=-\hat{V}\left(Y\right)+{\overset{\cdot }{W}}_{t}$$ (31)

with

$$\hat{V}\left(Y\right)=\frac{V\left[h^{-1}\left(Y\right)\right]}{\sigma \left[h^{-1}\left(Y\right)\right]}-\frac{1}{2}\sigma \prime \left[h^{-1}\left(Y\right)\right]$$ (32)

This is called the Lamperti transformation [see chapter 3.6 in (*45*)].

The Lamperti transformation allows for identifying the effective potential, $\hat{V}$, for the stochastic model (Eq. 30) subject to state-dependent stochastic disturbances given by $\sigma \left(X\right){\overset{\cdot }{W}}_{t}$. With this effective potential, Eq. 30 can be interpreted as a Brownian particle evolving within that potential whose fluctuations are just driven by a white noise, independently of the state *X*. In particular, even if the original potential *V* does not exhibit a well over a certain range of *X* values, the transformed potential can exhibit such local minimum. We talk then of noise-induced metastability in case of creation of an extra metastable state resulting from the interaction of the drift term with the diffusion term $\sigma \left(X\right){\overset{\cdot }{W}}_{t}$.

### Noise-induced metastability

By applying Eq. 32 to our stochastic droplet model (Eq. 15) in Pi-chamber, we obtain

$${\hat{V}}_{\lambda }^{\prime }\left(Y\right)=-\frac{V_{\lambda }^{\prime }\left(X\right)}{\sigma \left(X\right)}-\frac{1}{2}\sigma \prime \left(X\right),\;\text{with}\;Y=\int^{X}\sigma^{-1}\left(X\right)\;dX$$ (33)

in which *V*′_λ_(*X*) = −λ + *f*(*X*) + β*X*^−1/2^.

Note that the appearance of equilibria nontrivially depends on both the potential *V*_λ_ and the noise function σ. As a consequence of Eq. 33, the metastable states are explicitly given by solving ${\hat{V}}_{\lambda }^{\prime }\left(Y\right)=0$, namely, by solving

$$-V_{\lambda }^{\prime }\left(X\right)=\frac{1}{2}\sigma \prime \left(X\right)\sigma \left(X\right)$$ (34)

recovering this way Eq. 11. Thus, if one wishes to match the locations of these metastable states with let us say the modes of an empirical distribution (as in the “Pi-chamber empirical distributions versus Gibbs states” section), one needs to solve Eq. 34. As a consequence, this equation imposes constraints on the choice of the parameters involved in σ and *V*_λ_.

More precisely, we have that Eq. 34 rewrites as

$$\lambda -\tilde{A}X^{-1/2}+\tilde{B}X^{-3/2}-\beta X^{1/2}=\frac{1}{2}\sigma \prime \left(X\right)\sigma \left(X\right)$$ (35)

where $\tilde{A}$ and $\tilde{B}$ are defined in Eq. 17. By observing that $\sigma \left(X\right)=\sigma_{1}+\frac{\sigma_{2}-\sigma_{1}}{2}\left\{1+\text{tanh}\left[\kappa \left(X-X^{*}\right)\right]\right\}\to 0$ as *X* → ∞, one can get then, when κ ≫ 1 and *X* is sufficiently larger than *X*^∗^, good approximations of the solutions *X* to Eq. 35 by solving

$$\lambda X^{3/2}-\tilde{A}X+\tilde{B}-\beta X^{2}=0$$ (36)

We adopt this strategy for cases I and II of Pi-chamber empirical distribution versus Gibbs states.

The sink parameter β has a direct influence on the location of the metastable states and vice versa. One can invert Eq. 36 to find the value of β in terms of the activated mode *X*_c_

$$\beta =\frac{1}{X_{c}^{2}}\left(\lambda X_{c}^{3/2}-\tilde{A}X_{c}+\tilde{B}\right)$$ (37)

leading thus to Eq. 16.

The blue and red curves in Fig. 7 correspond to the left-hand side and right-hand side of Eq. 34, respectively, for the three case studies of the “Pi-chamber empirical distributions versus Gibbs states” section. The intersection of these two curves determines the location of the metastable states indicated by the dashed vertical lines for cases I, II, and III with the parameter configuration shown in Table 2.
